# Traditional knowledge of edible plants used as flavoring for fish-grilling in Southeast Guizhou, China

**DOI:** 10.1186/s13002-022-00519-7

**Published:** 2022-03-18

**Authors:** Jianwu He, Liping Peng, Wei Li, Jin Luo, Qiang Li, Hanyong Zeng, Maroof Ali, Chunlin Long

**Affiliations:** 1 College of Life and Environmental Sciences, Minzu University of China, Beijing, 100081 China; 2 College of Biology and Environmental Science, Jishou University, Hunan, 416000 China; 3 College of Mathematics and Statistics, Jishou University, Hunan, 416000 China; 4 College of Geography and Tourism, Hunan University of Arts and Science, Hunan, 415000 China; 5 College of Life Science, Anhui Normal University, Wuhu, 241000 China; 6 Key Laboratory of Ecology and Environment in Minority Areas (Minzu University of China), National Ethnic Affairs Commission, Beijing, 100081 China; 7 Key Laboratory of Ethnomedicine (Minzu University of China), Ministry of Education, Beijing, 100081 China; 8 Kunming Institute of Botany, Chinese Academy of Sciences, Kunming, 650201 China

**Keywords:** Edible plants, Traditional knowledge, Traditional agroecosystem, Ecosystem services

## Abstract

**Background:**

The local Dong people in Qiandongnan Prefecture, Guizhou Province, China, with rich biocultural diversity, have developed the traditional rice-duckweed-fish-duck agroecosystem (RDFDA) to support biodiversity conservation and to meet food and cultural needs. However, there is still not much research on traditional ecological knowledge (TEK) in this area. In particular, there is a lack of traditional knowledge of edible plants used by the Dong people as flavoring to grill fish (*Cyprinus carpio*) collected from RDFDA, which is extremely valuable in their traditional culture. The study focused on documenting plant species used in grilling fish and analyzing the status of its TEK.

**Methods:**

Twenty-one sampling points of three Dong minority villages in Qiandongnan were selected for the research. The local TEK associated with plant resources for fish-grilling was recorded through free listing and semi-structured interviews. Fidelity level (FL) and ethnoecological importance value (EIV) indicators were designed to determine the socioeconomic influence of TEK. The non-metric multidimensional scaling (NMDS) method was used to evaluate the differentiation of edible plant species distribution in dissimilar accessibility types.

**Results:**

A total of 430 people were interviewed about grilled fish, of whom 75% were men and 85% were farmers. Thirty-four edible plants were documented for fish-grilling in three Dong villages. They belong to 16 plant families, such as Apiaceae, and Asteraceae. The life forms included herbaceous (76%), shrubs (18%) and trees (6%). Leaves are the most commonly used part of for grilling fish, followed by aerial parts, and whole plants. Among these edible plants, *Allium hookeri*, *A. macrostemon* and *Houttuynia cordata* with the highest fidelity level (100%) were cited as edible plants for grilling fish by all informants. The NMDS showed different accessibility types of collection sites, with different importance values. Paddy rice field edge (2.03) has the highest value, followed by forest-farming ecotone (1.74), streamsides (1.71) and woodland (0.48).

**Conclusion:**

The purpose of this study was to investigate the traditional knowledge of edible plant materials used by the Dong people for grilling fish. The results demonstrate the strong connection between local people, the bio-environment and agroecosystem services. The survey and comparative analysis revealed that plant species with high FL values may be potential sources of natural flavors.

## Background

Globally, populations living in diverse habitats employ an abundance of edible plants to sustain their livelihoods. These edible plants carry certain social and cultural carrier functions, serving as a link to facilitate communication within communities, forming different styles of traditional ecological knowledge (TEK), and also contributing well to the maintenance of local biodiversity. It means, as a bridge point, biodiversity locates between ecosystem services and human well-being [1, 2]. Biodiversity in an ecosystem which could provide many ecosystem services such as food, fuel, shelter and building materials; stabilization and moderation of climate, floods, droughts, temperature extremes and wind forces; maintenance of genetic resources and the ability to adapt to change; and cultural, aesthetic and spiritual values, etc. [3].

In mountain areas of Southwest China, rice-fish farming is a traditional practice significant for the local environment [4, 5]. The rice-fish agriculture system in Southeast Guizhou is a standard sustainable agricultural system [6], which was recorded as globally important agricultural heritage systems (GIAHS) by the Food and Agriculture Organization (FAO) in 2011 [7]. The local population lives in a rich biocultural environment, manages and maintains their traditional complex agroecosystems. Actually, in Southeast Guizhou, the local ecological agriculture model of rice and fish (mainly refers to *Cyprinus carpio*, Cyprinidae) intercropping is subdivided into a few subtypes according to different habitats, based on recent research [6–8]. For example, the rice-fish-duck system, rice-fish-duck-forest system, and rice-duckweed-fish-duck agroecosystem (RDFDA) we proposed earlier [6–8]. RDFDA provides ecosystem services such as high genetic diversity of Kam sweet rice (*Oryza sativa* L.) phenotypes [5], wild edible plants and ethnic cultural values [9]. Additionally, it implements a long-term, sustainable use of resources as well as a way of farming that preserves traditional values.

The changes in human craft of cooking are related to technological change [10]. For ethnic groups with low limited food production, fire was a key tool for food processing and hunting in the wild [11, 12]. Previous studies have documented information on changes in fishing practices and folk fish cuisine [13, 14]. In the Southeast of Guizhou Province (Qiandongnan Prefecture), the Dong people manage the traditional rice-fish-duck agroecosystem in support of ecological and biodiversity conservation. During rice harvest, fish would be preserved with various edible plants for food and cultural uses (for large events such as weddings, funerals, and religious ceremonies) and for fish-grilling. Among them, traditional fish-grilling is one of the most impressive activities, preserving their ancient culture and their relationship with the environment.

China’s rural revitalization strategy was initiated by the central government of China in 2017 which often takes the village as the unit, and the farmer or the enterprise as the cell aiming to narrow the gap between urban and rural areas [15]. The Law of the People's Republic of China on the Promotion of Rural Revitalization clearly states that it is necessary to "develop industries with distinctive advantages" [16]. Additionally, the RDFDA in Southeastern Guizhou is also an influential local agricultural industry and a model for sustainable rural revitalization. This is capable of increasing the participation and benefit of local rural residents. Basically, the proposal complies with the Chinese government's strategy aim to promote organic links between small-scale farmers and agricultural development, with farmers as the main body and rural characteristics as supporting factor.

Despite the recognition of the importance of fish-grilling in Qiandongnan Prefecture, there are still few studies that focus on local Dong’s TEK in the region. Interestingly, the Qiandongnan area is a biodiversity hotspot in Guizhou Province with rich and diversified edible plants [9, 17]. Many of these species were collected from dissimilar accessibility locations such as paddy rice field edges, streamsides, forest-farming ecotone and woodland, and have not yet been cataloged or described. In particular, data is lacking on the perception of ethnoecological importance values and diversity of edible plants for fish-grilling in different accessibility types in the RDFDA. It is critical to local TEK that local people look for WEPs from disparate accessibility environments and learn how to use them. However, with the implementation of the urbanization strategy and the transformation of land use, the TEKs of Dong communities regarding the ecological aspects of species are often neglected. This results in the loss of significant information. Consequently, the traditional knowledge of edible plants used as flavorings for fish-grilling needs to be compatible with the local special microenvironment to be understood and described.

In view of this, we studied the Dong’s TEK of edible plants for fish-grilling, explored issues related to the intergenerational transmission of knowledge and sustainable utilization of resources in the region.

## Methods

### Study area

**Fig. 1:**
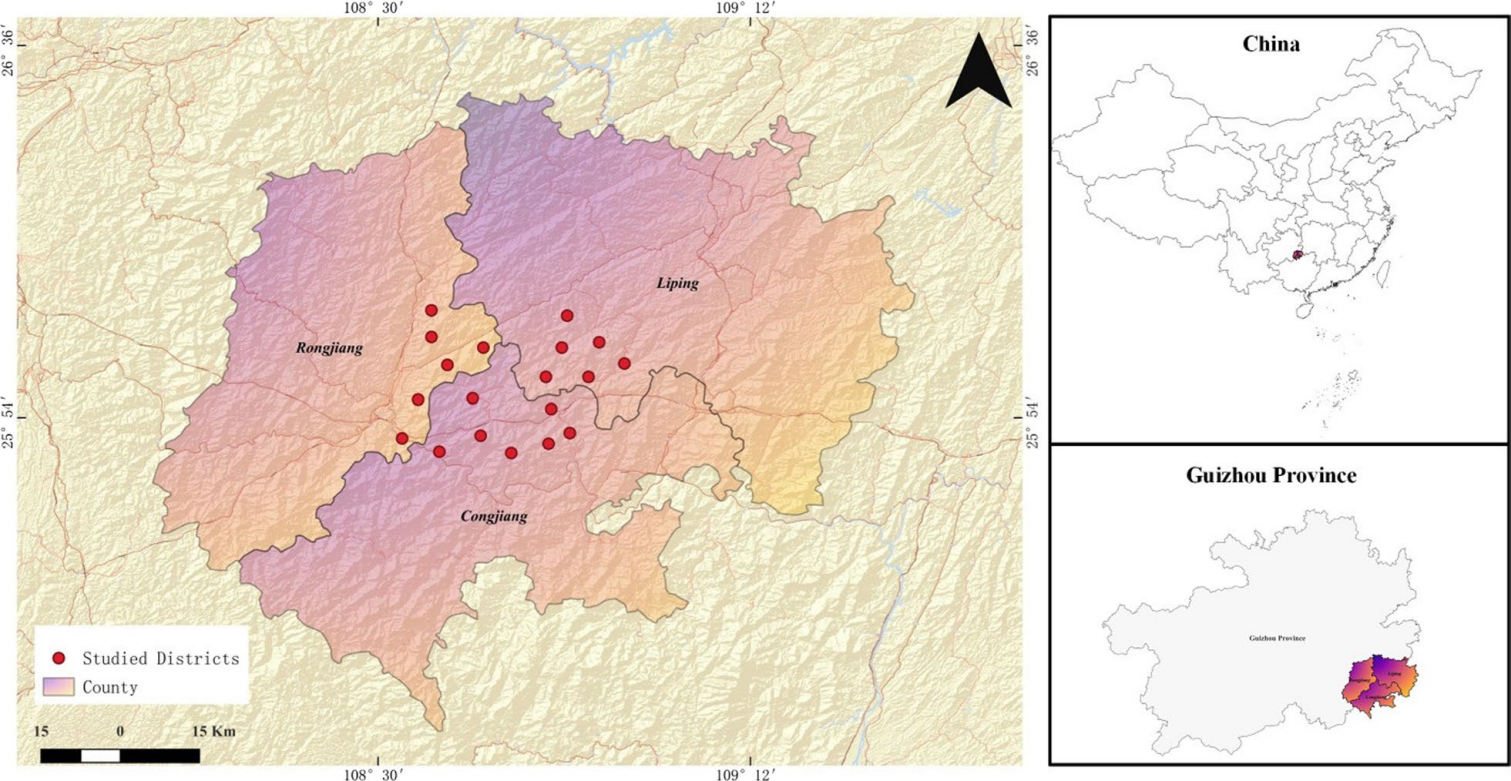
Geographic location of study area

The study was conducted in three villages in Qiandongnan, a prefecture in southeast of Guizhou Province, Southwest China (Fig. 1). Gaozeng and Shuangjiang villages are located adjacent to each other; Pingyang village is in the northwest of these two sites. According to official data, the area comprises medium and low mountains, hills and basins, with complex terrain and altitude variations from 1740 to 240 m. The climate of Southeast Guizhou is a subtropical monsoon humid climate, with a mean annual temperature of 14–18 °C, frost-free period is 270–330 days, the rainfall is 1000–1500 mm and the relative humidity is 78–84%. The majority of people in the study area belong to the Dong ethnic group. Based on figures published by the National Bureau of Statistics of China (NBSC, 2010), the population of Gaozeng, Shuangjiang and Pingyang villages is estimated at 15,987, 13,403 and 4,988, respectively (Table 1).

**Table 1:** Characteristics of the three Dong villages in Guizhou Province

Study villages	Location	Altitude (m)	Population			County
			Dong group	Other ethnic groups	Total	
Gaozeng	108° 56′ E–25° 48′ N	240–1035	15,987	1410	17,397	Congjiang
Shuangjiang	108° 55′ E–25° 57′ N	240–1218	13,403	5475	18,878	Liping
Pingyang	108° 20′ E–26° 17′ N	650–1704	5579	4988	10,567	Rongjiang

### Data gathering

The survey was carried out from September 2018 to April 2021 within three Dong villages (Table 1). Ethnobotanical walks were conducted jointly with community elders and part of respondents to check the availability, distribution, and accessibility of plants used for fish-grilling within the study area. In consultation with the district agricultural experts, administrators and community elders, a total of 21 sampling and informant sites are known to manage and maintain the traditional rice-duckweed-fish-duck agroecosystem (RDFDA), and were chosen for field investigations. This research was conducted after obtaining permission from the township committee and followed the ethical guidelines adopted by the International Society of Ethnobiology [18]. The edible plants used for fish-grilling and related local knowledge were collected through free listing and semi-structured interviews. Specifically, the number of plant species known by the inhabitants of each site documented with free listing. Semi-structured interviews as described by [19, 20] were conducted among 20–22 local informants per site who were identified by community members as knowledgeable about the traditional fish-grilling. Interviewees were asked the following questions related to the process of edible plant collection and eating method for fish-grilling in the region: (1) Would you mind listing some edible plants you often use to grill fish? (2) Where are you collecting them? (3) Could you tell us how to process them when fish-grilling? (4) Do you use them for other purposes? The questions appeared in the same order for all respondents. The interview was conducted in the Dong language and translated by the village leaders.

### Data analysis

The information of plant resources used for fish-grilling was recorded including the local names of species, habit, their uses in different forms, modes of administration, part(s) used and information concerning the edible value or relevance to local communities. The information forms were collected from three respondents for quantitative analysis. To determine the influence of socioeconomic factors, two different indicators of knowledge were used: (1) fidelity level [21], the fidelity level of each plant used was examined and based on combined use citation totals from all informants; (2) ethnoecological importance value [22] for one species by all informants. During the field observation period, plant specimens were collected, pressed and brought to the Jishou University Herbarium (JUH) for identification and authentication. The identification was made by consulting the *Flora of China* and The Plant List and by comparing with the authentic specimens in the JUH.

### Fidelity level

The fidelity level (FL) was calculated for the most frequently reported plant used for fish grilling with the highest FL value. The categories calculated for the *FL* percentage measure analysis are detailed in Table 2. Each plant use was added to the appropriate category prior to the calculation of the following formula:

$${\text{FL}} = N_{{\text{p}}} /N \times 100\%$$

where *N*_p_ is the total number of informants that independently cited a specific plant use and *N* is the total number of informants (N) that cited the plant for any use.

**Table 2:** Ethnographic data of local informants

Variables	Demographic categories	Population (*n* = 430)	Percentage
Gender	Male	325	75.58
	Female	105	24.42
Occupation	Farmer	367	85.35
	Trading	14	3.29
	Students	43	9.89
	Local officials	6	1.47
Age group	20–39	69	16.12
	40–59	195	45.41
	60 and older	170	39.47
Education status	Illiterate	139	32.22
	Primary	206	48.02
	Secondary	85	19.76
	Higher	5	1.20

### Ethnoecological importance value

Ethnoecological importance value: the index was calculated to understand the contribution of different accessibility types as sources of edible plant species for fish-grilling. Ethnoecological importance value was calculated by [22]:

$$EIV = \sum\limits_{x = 1}^{N} {(S)} \left( {\frac{nx}{{Nx}}} \right)$$

where *N* = total number of species in all accessibility types; *S* = Smith’s Saliency Index. This index measures the saliency level of a species by taking into account the frequency of mention of each edible plant species for fish-grilling by informants and the order it was mentioned by each informant. For this, it weighs the average of the inverse rank of a species across multiple lists where each list is weighted by the number of species in the list; *nx* = total number of individuals of species “x” found in one accessibility type; *Nx* = the sum of species “x” found in all accessibility types.

A non-metric multidimensional scaling (NMDS) was employed to assess the degree of distinctiveness of the distribution of WEP species in the different accessibility types. The NMDS was considered as an effective method to analyze species distribution at landscape scale, taking into account both environmental and biological factors [23–25]. NMDS was built on the free R 4.1.1 statistical program and performed in the *vegan* and *ggplot*2 package and based on Bray distance.

## Results

### Socio-economic characteristics of informants

A total of 430 informants were interviewed in the present study (Table 2). All informants practiced fish-grilling and also kept this traditional fish processing method due to the emotional connection with local farmers and the traditional RDFDA. Informants interviewed were mostly male (75%) and farmers (85%) are the most prominent occupations, followed by traders, students and local officials. More fish-grilling plants were reported by middle-age and old people (age > 40) than those by younger ones (20–39). And 32% of the respondents were illiterate (some even attended school but could not read).

### Diversity of edible plants for fish-grilling

**Fig. 2:**
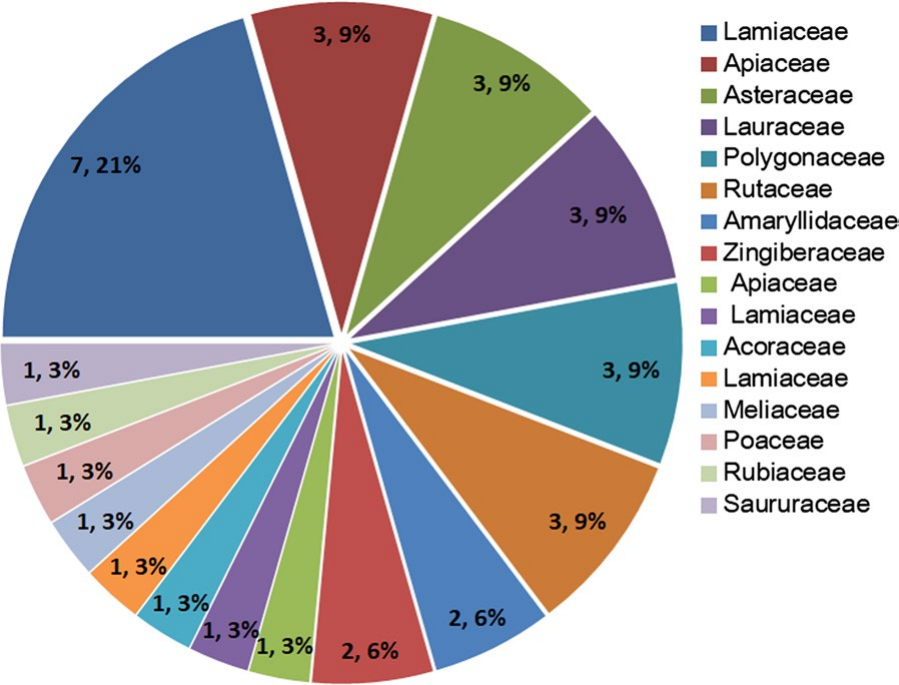
Use categories and the percentage of edible plants for grilling fish

As a result of our investigation of the three Dong villages, a total of 34 species of edible plants have proven to be useful for fish-grilling (Table 3). The results showed that the total richness was grouped into 16 botanical families (Fig. 2). The most commonly mentioned plant family was Lamiaceae (7 species, 21%), followed by Apiaceae, Asteraceae, Lauraceae, Polygonaceae and Rutaceae (3 species each), Amaryllidaceae and Zingiberaceae (2 species each). Plants from these 8 families contributed 76% of all species. All of these plants were native species.

**Table 3:** The inventory of Dong’s knowledge of edible plant species for grilling fish in the Southeast of Guizhou Province, China

S.#	Family	Species name	Chinese name	Dong (Kam) name	Life forms	Parts used	Fidelity level (%)
1	Acoraceae	*Acorus gramineus* Aiton	Jin qian pu 金钱蒲	Gekai	Herbaceous	Aerial parts	72
2	Lamiaceae	*Agastache rugosa* (Fisch. & C.A.Mey.) Kuntze	Huo xiang 藿香	Naobie	Herbaceous	Aerial parts	97
3	Amaryllidaceae	*Allium hookeri* Thwaites	Kuan ye jiu 宽叶韭	Maneng	Herbaceous	Whole plant	100
4	Amaryllidaceae	*Allium macrostemon* Bunge	Xie bai 薤白	Jiaodou	Herbaceous	Whole plant	100
5	Asteraceae	*Artemisia sieversiana* Ehrh	Da zi hao 大籽蒿	Yanxi	Herbaceous	Leaves	72
6	Lamiaceae	*Clinopodium chinense* (Benth.) Kuntze	Feng lun cai 风轮菜	Manao	Herbaceous	Leaves	83
7	Asteraceae	*Crassocephalum crepidioides* (Benth.) S.Moore	Ye tong hao 野茼蒿	Manengnong	Herbaceous	Aerial parts	95
8	Poaceae	*Cymbopogon citratus* (DC.) Stapf	Ning meng cao 柠檬草	Guanghe	Herbaceous	Leaves	72
9	Lamiaceae	*Elsholtzia ciliata* (Thunb.) Hyl	Xiang ru 香薷	Manao	Herbaceous	Leaves	56
10	Lamiaceae	*Elsholtzia kachinensis* Prain	Shui xiang ru 水香薷	Manong	Herbaceous	Leaves	56
11	Apiaceae	*Eryngium foetidum* L	Ci qin 刺芹	Gong	Herbaceous	Whole plant	67
12	Asteraceae	*Gynura bicolor* (Roxb. ex Willd.) DC	Hong feng cai 红凤菜	Masheng	Herbaceous	Aerial parts	73
13	Saururaceae	*Houttuynia cordata* Thunb	Ji cai 蕺菜	Sangfen	Herbaceous	Whole plant	100
14	Apiaceae	*Hydrocotyle sibthorpioides* Lamarck	Tian hu sui 天胡荽	Shalan	Herbaceous	Whole plant	82
15	Zingiberaceae	*Acorus macrospadiceus* F.N. Wei et Y.K. Li	Shan nai 山柰	Shajiang	Herbaceous	Whole plant	88
16	Apiaceae	*Ligusticum sinense* Oliv	Chuan xiong 川芎	Shuiqincai	Herbaceous	Aerial parts	73
17	Lauraceae	*Litsea cubeba* (Lour.) Pers	Shan ji jia 山鸡椒	Shuliuzi	Shrubs	Leaves; Fruit	67
18	Lauraceae	*Litsea mollis* Hemsl	Mao ye mu jiang zi 毛叶木姜子	Zhang;Cuiyouzi	Shrubs	Leaves; Fruit	65
19	Lauraceae	*Litsea pungens* Hemsl	Mu jiang zi 木姜子	Zhang;Cuiyouzi	Shrubs	Leaves; Fruit	66
20	Lamiaceae	*Mentha canadensis* L	Bo he 薄荷	Enqing	Herbaceous	Aerial parts	72
21	Lamiaceae	*Mentha spicata* L	Liu lan xiang 留兰香	Naowengzhen	Herbaceous	Aerial parts	89
22	Lamiaceae	*Ocimum basilicum* L	Luo le 罗勒	Gaiyansai; Mannao	Herbaceous	Leaves	62
23	Apiaceae	*Oenanthe javanica* (Blume) DC	Shui qin 水芹	Masi	Herbaceous	Stem	98
24	Lamiaceae	*Origanum vulgare* L	Niu zhi 牛至	Neng	Herbaceous	Leaves	83
25	Rubiaceae	*Paederia foetida* L	Ji shi teng 鸡矢藤	Jiaojing	Shrubs	Stem	52
26	Lamiaceae	*Perilla frutescens* (L.) Britton	Zi su 紫苏	Naoya	Herbaceous	Leaves	95
27	Polygonaceae	*Persicaria hydropiper* (L.) Delarbre	Shui liao 水蓼	Guge	Herbaceous	Leaves	72
28	Polygonaceae	*Polygonum lapathifolium* L	Suan mo ye liao 酸模叶蓼	Baiya	Herbaceous	Leaves	73
29	Polygonaceae	*Polygonum viscosum* Buch.-Ham. ex D. Don	Xiang liao 香蓼	Dun	Herbaceous	Aerial parts	95
30	Rutaceae	*Tetradium glabrifolium* (Champ. ex Benth.) T.G. Hartley	Lian ye wu yu 楝叶吴萸	Cuiyouzi,zheyi	Shrubs	Fruit	87
31	Rutaceae	*Tetradium ruticarpum* (A.Juss.) T.G.Hartley	Wu zhu yu 吴茱萸	Gongxiao; zheyi	Shrubs	Fruit	86
32	Meliaceae	*Toona sinensis* (Juss.) M.Roem	Xiang chun 香椿	Yeng	Trees	Leaves	62
33	Rutaceae	*Zanthoxylum bungeanum* Maxim	Hua jiao 花椒	Zhenyu	Trees	Leaves; Fruit	69
34	Zingiberaceae	*Zingiber striolatum* Diels	Yang he 阳荷	Niang; Xinxin	Herbaceous	Flowers	95

### The ethnoecological knowledge of edible plants

**Fig. 3:**
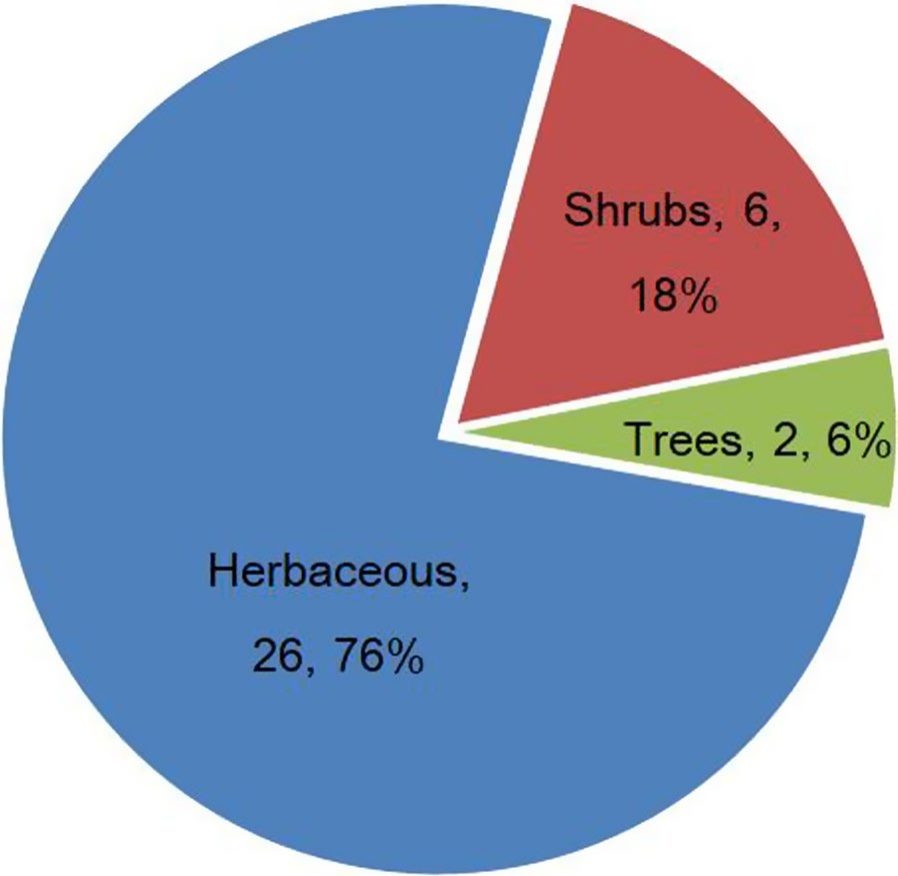
Life forms of edible plants for grilling fish

Based on the descriptions from informants, edible plants used for traditional grilled fish were generally collected between breaks in the glutinous rice harvesting, usually within 30 min of travel. Figure 3 shows that the leaves were the most popularly used part for fish-grilling and accounted for 11 species, followed by aerial parts (8 species), whole plant (6 species), leaves and fruits (4 species), fruits (2 species each) and flowers (1 species). These parts are generally flavorful and have good palatability, so locals choose these plant parts when grilling fish in the wild.

**Fig. 4:**
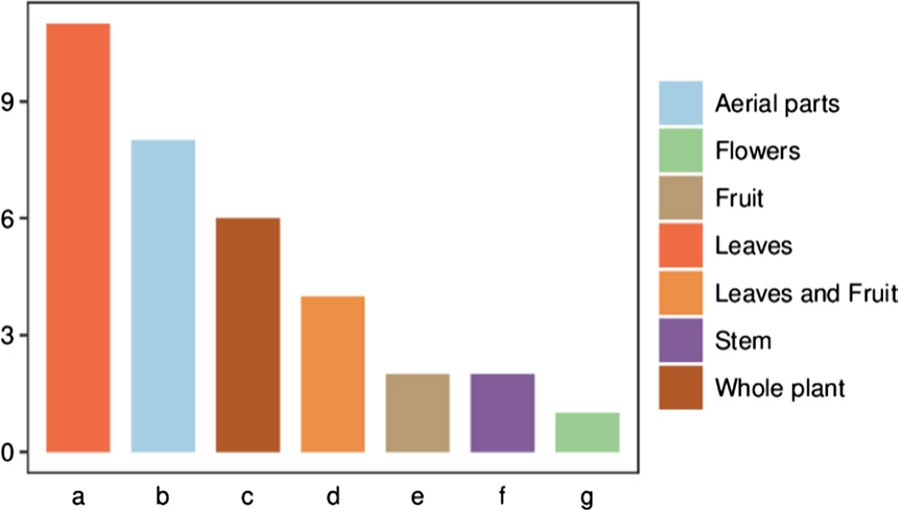
Parts used of edible plants for grilling fish

Figure 4 shows life forms of edible plants consumed by local Dong people for fish-grilling in southeast of Guizhou Province, China. Plants used for fish-grilling by the local Dong people had a diversity of growth forms including herbaceous, shrubby, and trees. Of the total 26 species are herbaceous plants account for a high percentage (76%), followed by shrubs with 6 species (18%) and trees with 2 species (6%) (Fig. 4).

**Fig. 5:**
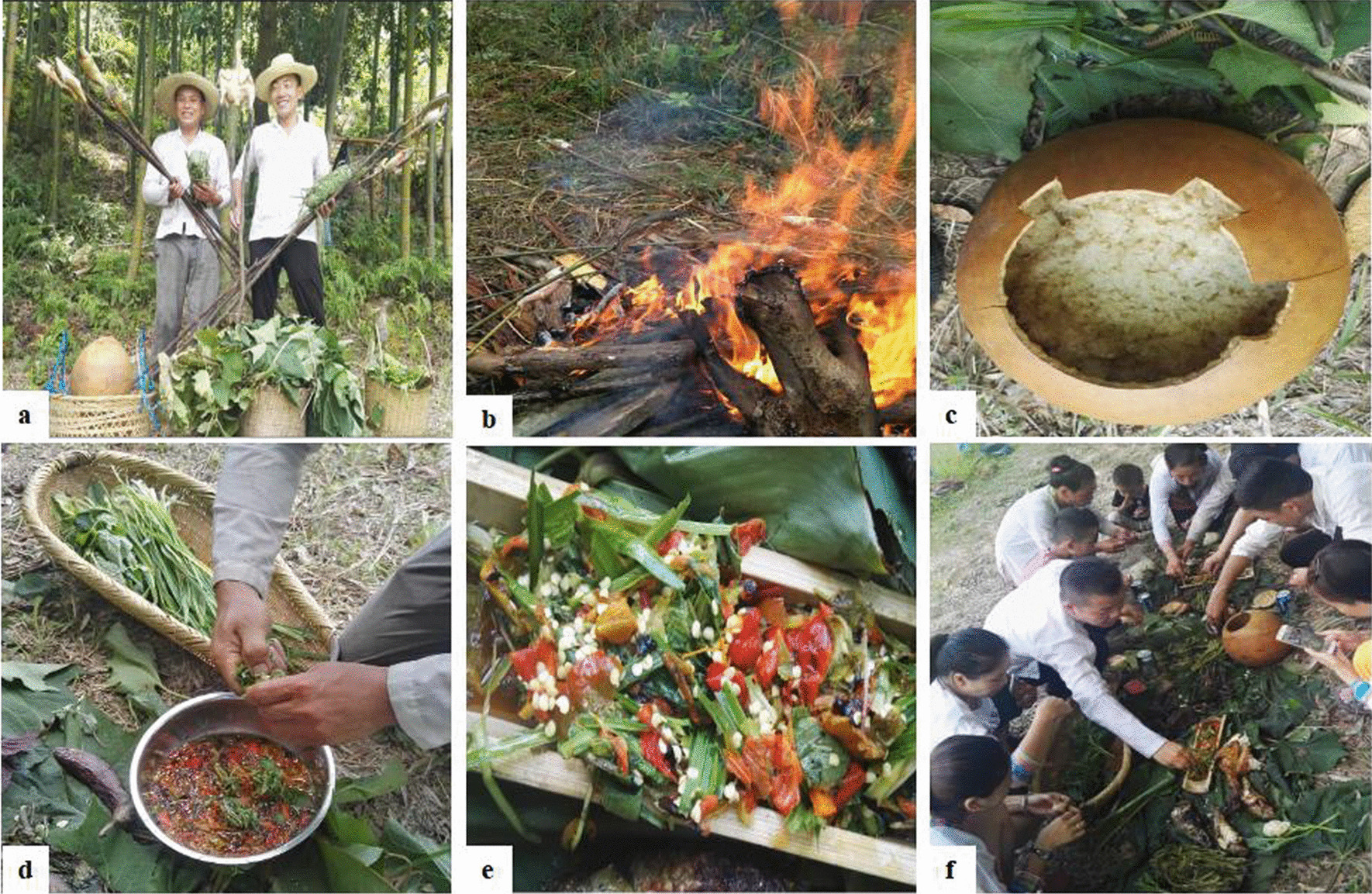
The procedure of fish-grilling (photographed by the first author). **a** Getting edible plants, fish and fuel materials ready, **b** Making fire and grilling fish, **c** Rice brought from home in a container made from *Lagenaria*, **d** Preparing edible plants, **e** Mixing edible plants in bamboo container, **f** Taking grilled-fish and rice when having lunch in the field

The Congjiang fish-grilling knowledge reflected the rich TEK of the locals. They usually fish while harvesting the rice. Some of these fish were grilled and served as lunch near the rice paddy, to reduce the time of returning home for dinner (Fig. 5), and some were taken home to wait for the next operation, making traditional grilled fish or for daily consumption [9]. Figure 5 shows a traditional fish grilling scene conducted by the Dong people of Xiaohuang Village on the forest-farming ecotone (gentle slope). Specifically, it consisted of the following 6 steps (Fig. 5):

- Preparation of edible plants, fish and fuel materials. Some of the edible plants are collected locally, while others (like chili peppers) are brought from home or the vegetable patch. Fish are live carp caught from rice paddies and kept in a nearby stream for a while. This allows debris such as silt in the gills to be exchanged through breath. Then they use long sticks to pass through the fish mouth and drain. The firewood was also collected nearby, mostly from trees and some small shrubs.
- Making fire and grilling fish. A suitable place to grill fish is usually found in the forest-farming ecotone area near the rice paddies. When the carps are cooked over a fire, locals do not seem to worry about burning the surface, as the burnt skin will be peeled off before eating.
- Homemade rice brought from home. In the morning, steamed rice is packed into a container made of *Lagenaria siceraria*. Rice will be taken from home as a staple food for lunch to the harvest site.
- Preparing edible plants. People need some seasoning to eat grilled fish. The collected edible plants are easy to handle and mix together to make a tasty fish-roasting sauce.
- Processed edible plants are stored in bamboo containers. Containers made of bamboo are used for storing food. It can be filled with condiments and steamed rice. It is easy for everyone to eat.
- Enjoying the lunch. In Southeast Guizhou, the harvesting of rice is usually carried out in the form of mutual aid groups composed of several families, which has long been a reciprocal mechanism. To celebrate the joy of paddy rice harvest, fish is grilled and shared. During the harvest, some activities will be organized, such as singing the Dong chorus [26] and tree climbing competitions.

### Fidelity level

In the study area listed in Table 3, the FL result for edible plants ranged from 52 to 100%. *Allium hookeri* (100%), *Allium macrostemon* (100%) and *Houttuynia cordata* (100%) were some of the species with high FL used as edible plants for fish-grilling. This would be a useful clue to track the role of these plants in improving the taste of grilled fish. Species with a low fidelity level were mainly used for broader applications.

### Ethnoecological importance values

As opposed to the general ecological ecosystem, the RDFDA’s environment and relevant variables are heavily influenced by human activities. Therefore, it is very difficult to study the spatial change as an ordinary ecological process [27]. Thus, it is feasible to study the distribution of species in this traditional agro-ecosystem by dividing the niche types appropriately based on participatory survey and local traditional ecological knowledge.

**Fig. 6:**
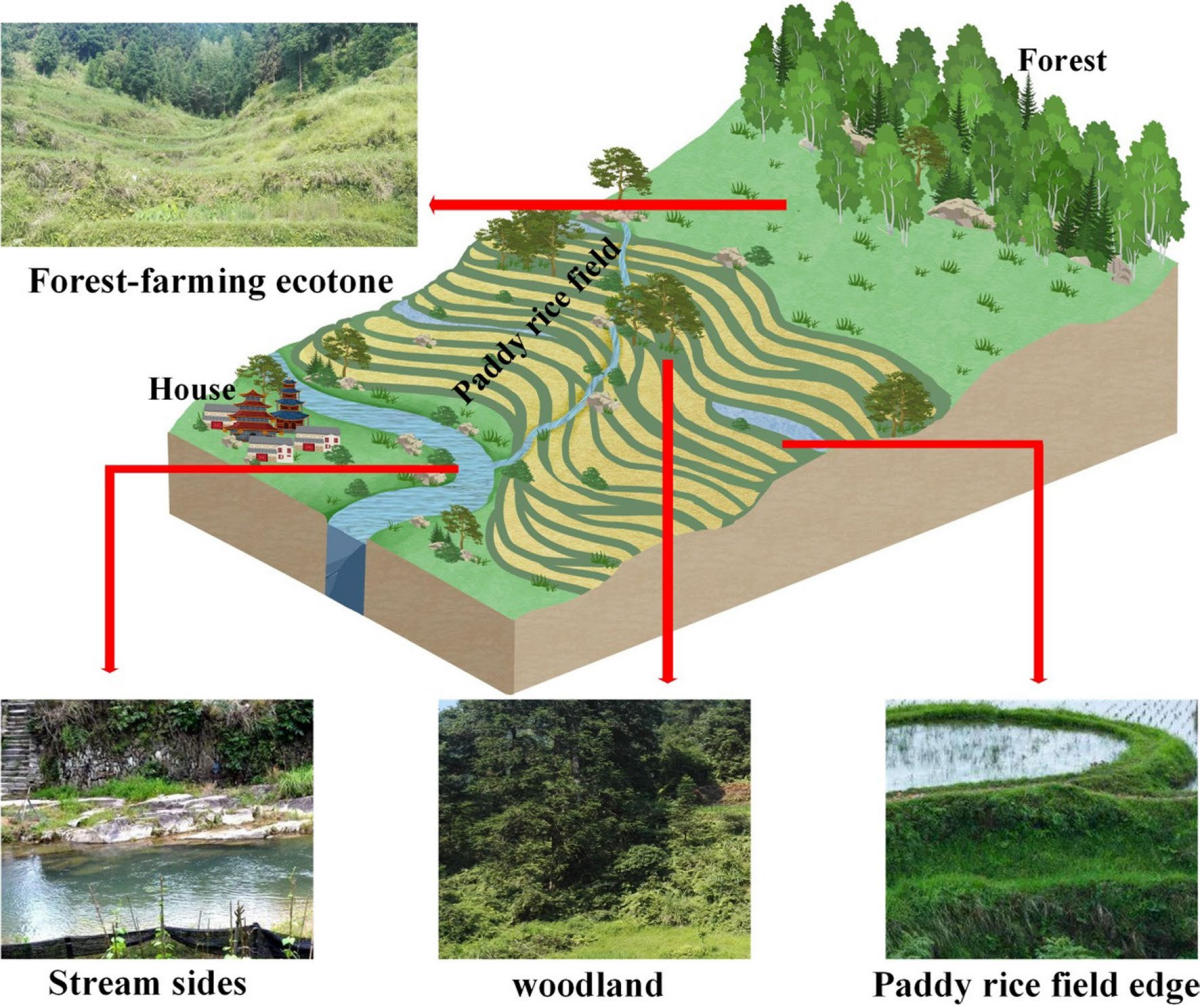
The edible plants used as flavoring for fish-grilling form dissimilar accessibility types

Within RDFDA there is high microenvironmental heterogeneity, including paddy rice field edges, stream banks, forest farming ecotones, and woodland with dissimilar access [28]. As shown in Fig. 6, the paddy rice field edges are ridges on the paddy field. Due to human disturbance, the vegetation in this area is dominated by annual herbaceous plants. Streamsides are common in the RDFDA. This small microenvironment can provide a continuous supply of water for the entire agricultural ecosystem. Forest-farming ecotone is the buffer zone between woodland, shrub secondary forest and paddy rice field, which is formed by artificial intervention such as mowing and cutting (the most suitable site for fish-grilling). Woodlands are artificially afforested lands close to paddy rice fields, and the understories are important collection habitats for edible plants.

Table 4 shows the ethnoecological importance values of the different accessibility types. Paddy rice field edge (2.03) appeared to have the highest ethnoecological importance, followed by forest-farming ecotone (1.74), streamsides (1.71) and woodland (0.48). Paddy rice field edge was dominated by *Mentha canadensis* and *Ocimum basilicum*, forest-farming ecotone by *Acorus macrospadiceus* and *Agastache rugosa*, streamsides by *Litsea cubeba* and *Gynura bicolor*, and woodland by *Mentha canadensis.* It was obvious that the closer the edible plants collection site was to the baked fish collection site, the greater its ethnoecological significance.

**Table 4:** The proportional abundance of edible plant species for grilling fish with different accessibility types

Species name	Species saliency score	Proportional abundance				Species saliency × abundance			
		Paddy rice field edge	Streamsides	Forest-farming ecotone	Woodland				
	*a*	*b*	*c*	*d*	*e*	*a* × *b*	*a* × *c*	*a* × *d*	*a* × *e*
*Acorus gramineus* Aiton	0.31	0.10	0.00	0.61	0.00	0.03	0.00	0.19	0.00
*Agastache rugosa* (Fisch. & C.A.Mey.) Kuntze	0.42	0.07	0.07	0.43	0.05	0.03	0.03	0.18	0.02
*Allium hookeri* Thwaites	0.55	0.05	0.02	0.11	0.02	0.03	0.01	0.06	0.01
*Allium macrostemon* Bunge	0.52	0.06	0.00	0.13	0.00	0.03	0.00	0.07	0.00
*Artemisia sieversiana* Ehrh.	0.33	0.09	0.00	0.33	0.00	0.03	0.00	0.11	0.00
*Clinopodium chinense* (Benth.) Kuntze	0.28	0.00	0.00	0.43	0.00	0.00	0.00	0.12	0.00
*Crassocephalum crepidioides* (Benth.) S.Moore	0.48	0.02	0.00	0.31	0.02	0.01	0.00	0.15	0.01
*Cymbopogon citratus* (DC.) Stapf	0.26	0.00	0.19	0.19	0.15	0.00	0.05	0.05	0.04
*Elsholtzia ciliata* (Thunb.) Hyl.	0.04	0.50	0.25	3.25	0.75	0.02	0.01	0.13	0.03
*Elsholtzia kachinensis* Prain	0.14	0.14	0.07	1.14	0.07	0.02	0.01	0.16	0.01
*Eryngium foetidum* L.	0.11	0.27	1.18	0.00	0.00	0.03	0.13	0.00	0.00
*Gynura bicolor* (Roxb. ex Willd.) DC.	0.29	0.21	0.59	0.14	0.00	0.06	0.17	0.04	0.00
*Houttuynia cordata* Thunb.	0.51	0.06	0.12	0.00	0.00	0.03	0.06	0.00	0.00
*Hydrocotyle sibthorpioides* Lamarck	0.38	0.08	0.42	0.00	0.00	0.03	0.16	0.00	0.00
*Acorus macrospadiceus* F.N. Wei et Y.K. Li	0.45	0.13	0.16	0.00	0.00	0.06	0.07	0.00	0.00
*Ligusticum sinense* Oliv.	0.42	0.12	0.33	0.12	0.10	0.05	0.14	0.05	0.04
*Litsea cubeba* (Lour.) Pers.	0.12	0.58	1.50	0.00	0.00	0.07	0.18	0.00	0.00
*Litsea mollis* Hemsl.	0.09	0.56	0.89	0.00	0.00	0.05	0.08	0.00	0.00
*Litsea pungens* Hemsl.	0.09	0.44	1.11	0.00	0.00	0.04	0.10	0.00	0.00
*Mentha canadensis* L.	0.16	2.00	0.63	0.31	0.38	0.32	0.10	0.05	0.06
*Mentha spicata* L.	0.32	0.50	0.19	0.00	0.09	0.16	0.06	0.00	0.03
*Ocimum basilicum* L.	0.14	1.57	0.43	0.00	0.00	0.22	0.06	0.00	0.00
*Oenanthe javanica* (Blume) DC.	0.41	0.27	0.07	0.00	0.00	0.11	0.03	0.00	0.00
*Origanum vulgare* L.	0.18	0.89	0.28	0.11	0.00	0.16	0.05	0.02	0.00
*Paederia foetida* L.	0.01	14.00	3.00	0.00	0.00	0.14	0.03	0.00	0.00
*Perilla frutescens* (L.) Britton	0.41	0.34	0.20	0.05	0.00	0.14	0.08	0.02	0.00
*Persicaria hydropiper* (L.) Delarbre	0.21	0.57	0.43	0.00	0.00	0.12	0.09	0.00	0.00
*Polygonum lapathifolium* L.	0.33	0.09	0.00	0.18	0.12	0.03	0.00	0.06	0.04
*Polygonum viscosum* Buch.-Ham. ex D. Don	0.47	0.02	0.02	0.19	0.17	0.01	0.01	0.09	0.08
*Tetradium glabrifolium* (Champ. ex Benth.) T.G. Hartley	0.36	0.00	0.00	0.06	0.03	0.00	0.00	0.02	0.01
*Tetradium ruticarpum* (A.Juss.) T.G.Hartley	0.19	0.00	0.00	0.11	0.16	0.00	0.00	0.02	0.03
*Toona sinensis* (Juss.) M.Roem.	0.15	0.00	0.00	0.40	0.07	0.00	0.00	0.06	0.01
*Zanthoxylum bungeanum* Maxim.	0.23	0.00	0.00	0.13	0.04	0.00	0.00	0.03	0.01
*Zingiber striolatum* Diels	0.41	0.00	0.00	0.15	0.12	0.00	0.00	0.06	0.05
Total ethno-ecological importance value						2.03	1.71	1.74	0.48

**Fig. 7:**
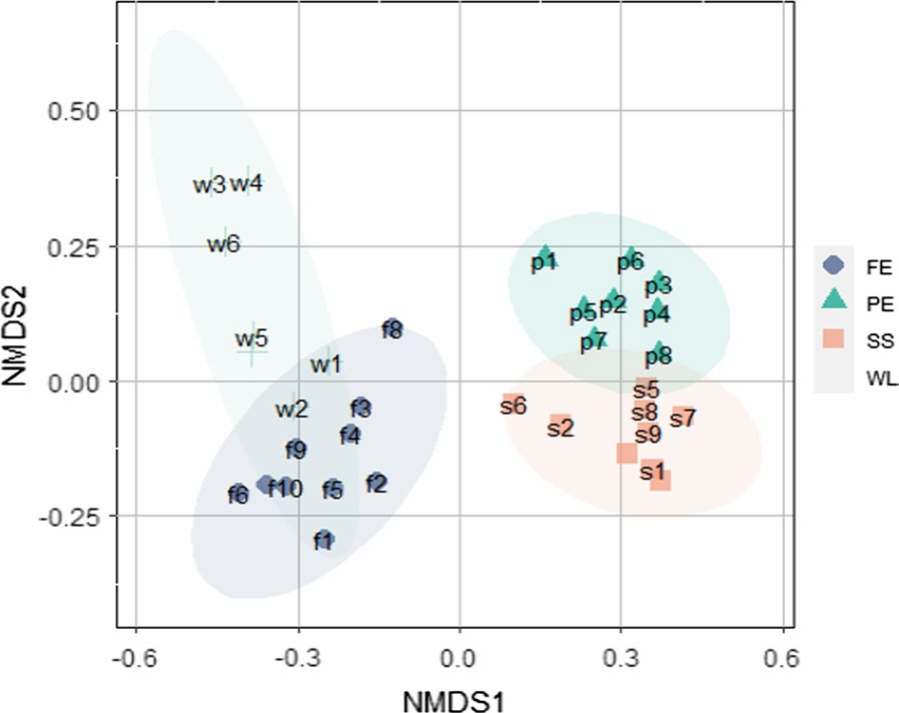
Non-metric multidimensional scaling (NMDS) showing the relationship between status of the groups (FE, Forest-farming Ecotone; SS, Streamsides; PE, Paddy rice field edge; WL, Woodland) (Stress = 0.08834)

The NMDS ordination of the 34 plant species by dissimilar accessibility types (Table 5). NMDS ordination showed that the horizontal axis (NMDS 1) divided the different accessibility types (Fig. 7). Group PE and SS showed higher NMDS 1 values. NMDS 1 was more positively correlated with accessibility of edible plants collection, which indicates that species of group PE and SS are more readily available. Some species of the lower NMDS 1 (w3, w4 and w6) values but higher NMDS 2 values included those that favor woodland and were only found in group WL, such as *Litsea cubeba*, *Litsea mollis*, and *Toona sinensis* (Fig. 7 and Table 5).

**Table 5:** The NMDS ordination of edible plant species for grilling fish by different accessibility types

S.#	Species name	Name	Group	NMDS1	NMDS2
1	*Agastache rugosa* (Fisch. & C.A.Mey.) Kuntze	f1	FE	− 0.2497164	− 0.29212915
2	*Clinopodium chinense* (Benth.) Kuntze	f2	FE	− 0.1509866	− 0.18809566
3	*Crassocephalum crepidioides* (Benth.) S.Moore	f3	FE	− 0.1810083	− 0.04823058
4	*Elsholtzia ciliata* (Thunb.) Hyl.	f4	FE	− 0.2012354	− 0.09685852
5	*Eryngium foetidum* L.	f5	FE	− 0.2338841	− 0.19875597
6	*Origanum vulgare* L.	f6	FE	− 0.4084669	− 0.20799353
7	*Paederia foetida* L.	f7	FE	− 0.3561403	− 0.19143242
8	*Tetradium glabrifolium* (Champ. ex Benth.) T.G. Hartley	f8	FE	− 0.1231253	0.09739216
9	*Tetradium ruticarpum* (A.Juss.) T.G.Hartley	f9	FE	− 0.3008836	− 0.12348621
10	*Zingiber striolatum* Diels	f10	FE	− 0.3220394	− 0.19667193
11	*Acorus gramineus* Aiton	p1	PE	0.3590456	− 0.16387644
12	*Allium hookeri* Thwaites	p2	PE	0.1889364	− 0.08175113
13	*Cymbopogon citratus* (DC.) Stapf	p3	PE	0.3113276	− 0.13505962
14	*Elsholtzia kachinensis* Prain	p4	PE	0.3723967	− 0.18553615
15	*Houttuynia cordata* Thunb.	p5	PE	0.3469459	− 0.01155976
16	*Acorus macrospadiceus* F.N. Wei et Y.K. Li	p6	PE	0.1000686	− 0.04268597
17	*Oenanthe javanica* (Blume) DC.	p7	PE	0.4164526	− 0.06612494
18	*Persicaria hydropiper* (L.) Delarbre	p8	PE	0.3434208	− 0.05245737
19	*Polygonum lapathifolium* L.	p9	PE	0.3505306	− 0.09577703
20	*Allium macrostemon* Bunge	s1	SS	0.1579101	0.22577891
21	*Gynura bicolor* (Roxb. ex Willd.) DC.	s2	SS	0.2847046	0.14629648
22	*Hydrocotyle sibthorpioides* Lamarck	s3	SS	0.3685032	0.17971453
23	*Mentha canadensis* L.	s4	SS	0.3673959	0.12746643
24	*Mentha spicata* L.	s5	SS	0.2309077	0.13087931
25	*Ocimum basilicum* L.	s6	SS	0.318139	0.22380699
26	*Perilla frutescens* (L.) Britton	s7	SS	0.2496252	0.0757549
27	*Polygonum viscosum* Buch.-Ham. ex D. Don	s8	SS	0.3701648	0.04940446
28	*Artemisia sieversiana* Ehrh.	w1	WL	− 0.2442443	0.03655016
29	*Ligusticum sinense* Oliv.	w2	WL	− 0.3082521	− 0.04632943
30	*Litsea cubeba* (Lour.) Pers.	w3	WL	− 0.4611695	0.36688339
31	*Litsea mollis* Hemsl.	w4	WL	− 0.3932469	0.37030643
32	*Litsea pungens* Hemsl.	w5	WL	− 0.3836315	0.08423934
33	*Toona sinensis* (Juss.) M.Roem.	w6	WL	− 0.4338251	0.2580351
34	*Zanthoxylum bungeanum* Maxim.	w7	WL	− 0.3846193	0.05230323

## Discussion

The Dong people of Qiandongnan area operated the famous rice-fish agroecosystem and had a complete set of traditional knowledge of managing and administering the ecosystem (RDFDA). Our study revealed the TEK of 34 edible plants main collection from 4 dissimilar accessibility environments in this ecosystem that were used by locals for traditional fish grilling. Results showed that (1) Edible plants were irreplaceable in shaping TEK and productive landscape; (2) The distribution of these resources represented their different ethnoecological importance and performed important system service functions; (3) Ethnobotany of native edible plants, compared with other regions; (4) Intergenerational transmission of traditional knowledge related to this type of resource use was dramatically affected by changes in production methods; (5) Edible plants may contain important flavor substances that were worthy of further exploration; and (6) It was suggested that the sustainable use and development of edible plants resources and their productive landscapes requires a multidisciplinary and interdisciplinary perspective of systems integration.

### Edible plants were irreplaceable in shaping TEK and productive landscape

Studies showed that the use of edible plants was closely related to traditional culture creation [29–31]. Consistent with the theory of cultural change of Steward, culture should be viewed in the context of the entire natural and social environment in which human beings live. Therefore, in the face of the objective impact of productivity change on the collection of edible plants’ traditions, it was necessary to establish an ecological field perspective. Whether we admit it or not, traditional culture is in constant flux. An innovation in the study area was the transformation of residential houses into and other rural revitalization projects, one of which was to combine spice plants collected from the surrounding area with activities such as grilling fish and meat. This trend was concerning with the potential changes in land use, rice paddies, ditches, agroforestry and woodlands would all change to some degrees. It is necessary to preserve local productive landscapes and related local knowledge.

### Ethnoecological importance and systemic service functions of edible plants species

Both the field survey data and ethnobotanical walk observations indicated that traditional baked fish had been and continued to be an important part of Dong traditional culture. On the basis of the NMDS analysis, different sample collection environments representing different accessibility types could be visually distinguished. In paddy rice fields, forest-farming ecotone, streamsides and woodlands edible plants occupy their own ecological niches. They are significant services, providing a distinctive dietary supplement to the local population. It was important to note that the ethnoecological importance values of these species reflected their importance for regional agroecosystems maintenance. TEK related to these species and resource use had an irreplaceable role in maintaining rice-fish symbiotic systems and biodiversity. Due to rural revitalization in China, the local tradition of fish grilling had evolved into a special experience.

### Ethnobotany of native edible plants, compared with other regions

The species diversity of edible plants, especially WEPs, is obviously closely related to the local vegetation cover. Therefore, it is not enough to measure the richness of the traditional knowledge of the edible plant resources used as flavoring for fish-grilling in Southeast Guizhou by species diversity alone. Compared to other regions in terms of the use of edible plants, this study found that the Dong people’s knowledge of edible plants for fish-grilling (including uses, processing methods, and acquisition technology) was significantly different from the others.

An ethnobotanical study conducted to catalog and describe edible plants of a region as a whole [32–34]. Few case studies were as precise as this study for a particular dietary culture. In terms of processing, unlike other cases in which edible plants were brought back to the kitchen for cooking [35], the WEPs collected in this study were washed nearby and taken directly within the site to harvest rice and fish. It was found that the Dong people were skilled at collecting WEPs, quickly gathering the necessary plants for fish-grilling from four dissimilar environments of the RDFDA in less than 15 min. These differences illustrate the wealth of TEK acquired by the Dong people as a result of long-term management of the local ecosystem.

### Elements influencing the TEK intergenerational transmission related to edible plants resources use

The methods of gathering edible plants for fish grilling are common skills among the locals. Socio-economic characteristics of informants showed varying degrees in terms of occupation, age, and educational attainment. It was believed that there might be irreversible impacts on the intergenerational transmission of TEK in relation to edible plants resources use. This is because local young people migrate for work and schooling. It was found that current edible plants in different habitats were mostly collected by middle-aged and older adults > 40 years old, which is consistent with Yeşil’s study [36]. In the rural revitalization strategy implemented by the Chinese government, traditional culture inheritance is of paramount importance. It is essential to conserve and inherit TEK in the context of sustainable local resource use, and develop industries suitable for the coordinated development of local ecology-society-culture.

### Edible plants may contain important flavor substances

Results suggested the palatability factor was a common consideration for locals when choosing plants to use as an accompaniment for grilled fish. *Allium hookeri*, *Allium macrostemon* and *Houttuynia cordata* showed 100% FL value, according to available studies [37–42]. These findings fully corroborated the availability hypothesis of such composite plants selected by the Dong people. There is a lack of direct medical or pharmacological mechanism evidence to support the hypothesis that these species may benefit human health. These key aromatic compounds, however, can be followed up as potential sources of natural flavors for further study.

### Sustainable management of edible plants resources and agroecosystems requires a multidisciplinary perspective

The main results of these studies were based on classical ethnobotany, sociology, ecology and statistics. However, sustainable management of agroecosystems is a complex and systematic project. This paper enables people to understand the TEK related to the RDFDA edible plant resources, which were collected by Dong people in Qiandongnan, Guizhou Province.

A multidisciplinary approach must be used to explore the mechanisms for local practices and sustainable ecosystem maintenance, including human geography, natural product chemistry and pharmacology, and other cross-disciplinary research methods.

## Conclusions

This is an ethnoecological study on the traditional knowledge of the Dong people in Qiandongnan area who collect edible plants for grilling fish. It reported 34 edible plant species gathered from four dissimilar accessibility environments and consumed by the locals. These species are distributed in different habitats of the RDFDA and each contributes different ethnoecological values. Some of the species that showed the highest FL values for improving the taste of grilled fish included *Allium hookeri*, *A. macrostemon* and *Houttuynia cordata*. The results demonstrate the strong bond between the Dong people and nature, as well as the integrated nature-society-culture influence on TEK related to edible plants resource use. With the implementation of the rural revitalization strategy, the traditional livelihoods and land use of the Dong people in Qiandongnan area have changed. This study suggests effective measures to reduce the impact of TEK intergenerational transmission related to edible plant use. It calls for multidisciplinary knowledge integration to enhance the sustainable management of local natural resources and agroecosystems. Finally, the survey and comparative analysis revealed that species with high FL values may be potential sources of natural flavors, which is subject to follow-up study.

## Data Availability

The data for this study may be availed upon request.
